# Influencing factors of smartphone addiction in the era of artificial intelligence: based on the perspective of home-school

**DOI:** 10.3389/fpsyg.2026.1770457

**Published:** 2026-05-08

**Authors:** Jianxiang Fei, Yingyu Zhang

**Affiliations:** 1 Faculty of Education, Qufu Normal University, Qufu, China; 2 School of Business, Jiangsu Ocean University, Jiangsu, China

**Keywords:** artificial intelligence era, home-school perspective, influencing factors, smartphone addiction, undergraduates’ sustainable development

## Abstract

In the era of artificial intelligence, undergraduates have the characteristics of convenience and time flexibility in the use of smart phones, and their addiction is directly related to the sustainable development of students “physical and mental health. Family and school are the main fields of their life and learning, so exploring the dependence of undergraduate students on smartphones from the dual perspectives of home and school undoubtedly has important social responsibility significance. We use *t*-test, one-way analysis of variance and stepwise regression method to analyze the data of 556 undergraduates, and explore the differences of undergraduates” smartphone addiction, the influencing factors from the perspective of home-school and the interaction effect between home and school. The study shows that there are significant differences in gender, number of children in the family, and grade among undergraduates’ smartphone addiction. Social capital within and outside the family, family embodied and objective cultural capital, and student support have a significant negative impact on undergraduates “smartphone addiction. Family economic capital, family institutionalized capital, teacher support and independent opportunities have no significant impact on undergraduates” smartphone addiction. Social capital within and outside the family has a mutual substitution effect with student support, and family embodied and objective cultural capital has a mutual promotion effect with student support. Therefore, we should pay attention to the classification of smartphone addiction, make good use of family capital to regulate smartphone addiction, and create student support.

## 1 Introduction

The 57th Statistical Report on the Development of Internet in China shows that by December 2025, the number of mobile Internet users in China is 1.1205 billion, accounting for 99.6% of all Internet users, which shows the widespread use of mobile phones (CNNIC, 2026). At the same time, smartphones have become the standard equipment for undergraduates, and are widely used in social networking, online entertainment, etc. (Bonneville and Riddell, 2023). However, undergraduates’ use of smartphones has gone beyond the normal situation, forming the phenomenon of smartphone addiction, causing problems such as academic burnout (Liu et al., 2026) and seriously affecting the sustainable development of students’ physical and mental health. This is contrary to the Outline of the 14th Five-Year Plan for Economic and Social Development and Long-Range Objectives Through the Year 2035 of the People’s Republic of China, which puts emphasis on the development of students’ physical and mental health, and therefore the issue of smartphone addiction among undergraduates needs to be addressed properly.

Previous studies have examined the factors influencing smartphone addiction among undergraduates from the perspective of the smartphone factors, individual user characteristics and psychological factors. For instance, Mobile phone use patterns influence mobile phone addiction (Hao et al., 2019). Childhood trauma and perceived stress positively affect smartphone addiction among undergraduates (Li et al., 2025). Neuroticism positively affects smartphone addiction among undergraduates, while psychological capital negatively affects it (Yan et al., 2024). But few have examined the role of the family and school on undergraduate smartphone addiction. According to Family Capital Theory, families can influence undergraduates’ thinking, habits and behaviors in a certain field through their various capital operation, and parental involvement in education and financial security for learning support affects undergraduates’ behaviors too. Meanwhile, the individual-environment matching theory suggests that schools influence students’ daily behaviors, such as smartphone dependency, through the pervasive campus climate. This paper explores the different analysis of Chinese undergraduates’ smartphone addiction from demographic variables, analyzes the influencing factors of Chinese undergraduates’ smartphone addiction from the perspectives of family capital and school environment, and tries to propose effective strategies to curb Chinese undergraduates’ smartphone addiction from the aspects of family capital factor and school climate.

## 2 Literature review

### 2.1 Research on the connotation and causes of smartphone addiction

With the rapid development of intelligent technology, using mobile phones has become a common phenomenon. Reasonable use of mobile phones can bring convenience to life and work, but the current use of mobile phones has exceeded the reasonable degree, resulting in the phenomenon of addiction to mobile phones, which is called mobile phone dependence in relevant studies. Mobile phone dependence, also known as mobile phone addiction or abuse, refers to an individual’s addiction to various activities mediated by mobile phones, resulting in a strong and sustained sense of desire and dependence on mobile phone, and leading to significant impairment of social and psychological functioning (Yen et al., 2009). Compared with traditional mobile phones, smartphones are deeply infatuated by undergraduates due to their outstanding features such as powerful functions and user-friendly design, resulting in reduced sleep quality, degraded academic performance, and other diseases such as mobile phone addiction, which eventually become smartphone addiction.

The causes of smartphone addiction come from multiple aspects, including factors related to smartphones, individual user characteristics, and user psychology. Specifically, from the perspective of the inherent factors of smartphones, firstly, they can provide rich and personalized content, making undergraduate students infatuated or even addicted; Secondly, the functions of smartphones are becoming increasingly powerful and convenient, such as communication, social functions, etc. which make students gradually become dependent on smartphones due to long-term use (Nahas et al., 2018). From the perspective of individual user characteristics, demographic factors such as gender and educational background also affect smartphone addiction and show significant differences. Studies have found that female smartphone addiction tendency is higher than male (Demirci et al., 2014); From the perspective of users’ psychological needs, undergraduates rely on smartphones to meet their social needs and entertainment needs (Hsiao et al., 2016). In addition, scholars have also explored smartphone addiction in terms of environmental factors, personal factors. Studies have shown that parents’ long-term use of smartphones (Liu et al., 2019), smartphone use of surrounding groups (Wang et al., 2022), personality traits (Kheradmand et al., 2023), and students’ loneliness (Kürtüncü et al., 2021) all have a great impact on undergraduates’ smartphone addiction.

### 2.2 Studies on household capital

Household capital, which originates from social capital theory, refers to the capital acquired by individuals due to ascribed factors like family origin, also known as predisposing blood social capital. To trace the origin of household capital, we must first clarify the origin of social capital theory. Theodore W. Schultz, an American economist, proposed the theory of “human capital” in the 1960s, which expanded the concept of “capital” in classical economics to a broader sense. In 1967, Blau and Duncan proposed the “status realization model” in their book The American Occupational Structure, pointing out that there is a significant connection between education acquisition and family background, which to some extent influenced the emergence of social capital theory. In the 1970s, Pierre Bourdieu and James Samuel Coleman included social relations and network structure in their analysis and proposed the social capital theory. Since then, the research field of capital has expanded from economics to education and sociology. Scholars have found that economic, cultural and social resources within the family have an important impact on education, career and social stratification, and put forward the concept of family capital. Coleman, an American scholar, believes that family capital is divided into physical capital, human capital and social capital, while French scholar Bourdieu pointed out that family capital contains family economic capital, family social capital and family cultural capital (Tan et al., 2023), and can provide various useful resources for students’ education, which has a profound influence on students.

The profound impact of family capital on educational research is evident in published papers. Related researches mainly focus on academic performance, education attainment, college student employment, educational equity, and college student campus behavior. Specifically, from the perspective of academic performance, studies mainly focus on the significant influence of family capital on the acquisition of academic achievement (Wei et al., 2022); as for graduate employment, family capital has a significant influence on graduates’ career development (Ion, 2023); with regard to educational equity, relevant studies have explored the impact of different types of family capital on access to higher education opportunities (Tan et al., 2023).

### 2.3 Studies on school climate

School climate is a relatively stable and enduring feature of the psychosocial environment that individuals or group members experience in school and that influences their psychology and behavior, usually reflecting the organizational structure, teaching and learning practices, norms, goals, values and interpersonal relationships of the school. As a complex, multidimensional concept, school climate can be analyzed in terms of organizational climate, social system environment and psychosocial climate. It has been found that school climate includes participation of peer support in school activities, teacher support, students’ autonomous opportunities and a sense of belonging to the school.

The impact of school climate on students is multifaceted, including adolescent behaviors, school adaptability and learning engagement. In terms of adolescent behaviors, it was found that a positive school climate predicted adolescent misbehavior (Zhang and Wang, 2020); From the perspective of school adaptability, school climate can effectively reduce boredom and loneliness (Yu et al., 2019), so as to enhance school adaptability. As for learning engagement, a positive school climate was effective in promoting students’ investment in learning time and energy (Zysberg and Schwabsky, 2021).

According to self-determination theory, when students are lonely and basic psychological needs such as relational needs are not met, they feel frustrated and anxious, and turn to smartphone dependency to seek psychological satisfaction, while when students are engaged in learning, the satisfaction of psychological needs such as self-directed and competence needs motivates students to actively seek self-growth and discourage risky behaviors such as smartphone addiction. In addition, a positive campus atmosphere significantly avoids misbehavior, which has a suppressive effect on smartphone addiction. In conclusion, school climate can have an impact on smartphone addiction.

### 2.4 Summarize

Scholars have conducted systematic research on the problem of smartphone addiction from the aspects of current situation, causes and adverse consequences and formed a relatively unified understanding. Studies on the factors influencing smartphone addiction are relatively mature, but there are still some deficiencies in the existing studies: firstly, studies on the factors influencing smartphone addiction did not take family factors into account, especially lacking in systematic analysis of the relationship between family economic, social, cultural capitals and smartphone addiction; secondly, few studies focus on the impact of school climate on smartphone addiction, specifically, the impact of teacher support, student support and autonomy opportunity on smartphone addiction; thirdly, existing studies pay little attention on the joint impact of family capital and school climate on smartphone addiction. It is well known that individual socialization begins at home and forms at school, and undergraduates’ smartphone addiction is part of individual socialization and is therefore largely influenced by their families and schools. The main contributions are to clarify the influence of family capital and school climate on smartphone addiction, with a view to proposing targeted measures to regulate smartphone addiction from both family and school perspectives.

## 3 Research hypotheses

### 3.1 The effect of family cultural capital on smartphone addiction

Social Reproduction Theory claims that children’s habitus of thinking mode and behavior in the family field is nurtured by family cultural capital. The idiosyncrasy that children acquired in family life will be projected on the future individual study and life with a profound and long-lasting effect. At the same time, according to Bourdieu, cultural capital can exist in three forms: in the embodied state, in the objectified state, and in the institutionalized state (Bourdieu and Richardson, 1986). Embodied cultural capital is the long-lasting dispositions and cognitive consciousness internalized in the mind and body. The objectified cultural capital is embodied in the cultural entity and cultural environment possessed by the family. The institutionalized cultural capital is embodied in the qualifications or diplomas that parents possessed.

The large body of research suggested that family cultural capital can affect students’ behavior at school through the habitus of cognition and mindset awareness such as the focus on their academic study and learning expectation. The connotation of different types of family cultural capital are different. To be specific, the measurement of parents’ cognition and priority to education, family book collection, and the highest academic qualifications among parents. Long-term exposure to such cultural atmosphere can imperceptibly cultivate and shape children’s many characteristics and temperament, such as academic attitude and spare time arrangement, so that they can avoid smartphone addiction in terms of time, energy and psychological needs satisfaction. A research found that parents with higher family institutional cultural capital emphasized on their children’s education planning (Kirchsteiger and Sebald, 2010). Families with high levels of embodied cultural capital mean that parents are more concerned with transmitting education philosophy to their children (Xie and He, 2023). Families with higher objectified cultural capital mainly possess more book collection and other cultural objects, which is essential for creating a cultural atmosphere for their children (Yu et al., 2022). In this way, students’ good behavior at school will be shaped through the management of spare time, devotion in learning, learning expectations, which in turn acts as a disincentive to smartphone addiction.

Based on Social Reproduction Theory and the finding of relevant empirical studies, this study attempts to probe into the influence of family embodied cultural capital, family objectified cultural capital, and family institutionalized cultural capital on undergraduate students’ smartphone addiction, and proposes the following hypotheses.

*H1a*: There is a negative correlation between family embodied cultural capital and undergraduates’ smartphone addiction.

*H1b*: There is a negative correlation between family objectified cultural capital and undergraduates’ smartphone addiction.

### 3.2 The effect of family economic capital on smartphone addiction

There exist two different views on the effect of family economic capital on college students’ performance. First off, a significant positive correlation between family economic capital and college students’ performance can be proved, which means the higher the family economic capital, the better the performance of college students. Scholars who hold this view believe that the higher household property and income would guarantee the sufficient nutrition, a safe socialized environment and abundant learning facilitation, and students can also enjoy a higher level of education and guidance (Zhao et al., 2023). Secondly, there is a significant negative correlation between family economic capital and college students’ academic performance. The higher the family economic capital, the worse the college students’ academic performance. Scholars who hold this view believe that although families with sufficient economic capital can provide better material conditions, their time to accompany children will be crowded out. Subsequently, parents will spend less time on parent-child communication and communication with their children from the perspective of spiritual conditions. And the negative impact of shortened companionship on a child’s development can far outweigh material supporting (Valle Arias et al., 2016). According to the Theory of Income Effect and Substitution Effect, this study argues that the increase of parents’ working hours leads to less companionship and communication with their children. The negative impact on children’s cognitive ability far exceeds the material support brought by the increased income. As a result, children’s lack of correct perception of smartphones and inefficient communication caused their psychological needs to be unmet. Resorting to negative behaviors makes undergraduates’ more addiction on smartphones.

The undergraduates’ smartphone addiction is not a problem that can be merely coped with material abundance. To a certain extent, parents’ companionship and communication are more essential than material abundance. Based on the above theories and existing empirical studies, this study proposes the following hypothesis:

*H2*: Family economic capital has a positive impact on college students’ smartphone addiction.

### 3.3 The effect of family social capital on smartphone addiction

Family social capital is a social resource structure that exists in interpersonal network and can be used as assets to help individuals achieve their goals. Coleman classified it into social capital within the family (also known as intergenerational closure) and social capital outside the family (also known as social closure). The former refers to the relationship between parents and children, mainly including parents’ attention, support and communication for children’s education. The latter refers to other social relationships of parents and children, including contact with teachers and so on. Existing studies have shown that family social capital has a significant impact on the development of children’s cognitive aptitude (Kai, 2022). Therefore, family social capital can affect undergraduates’ correct cognition of smartphone use, thus effectively avoiding smartphone addiction to a certain extent. Meanwhile, parents with higher social capital within the family can effectively help their children to make a rational career development planning through educational participation and educational enhancement, so that they can work hard to strive for the success of each stage in school, enrich the academic life of college students (Jaiswal and Choudhuri, 2017), and avoid the mobile phone addiction due to idleness. Because of the social capital outside the family, the parents can communicate with their children’s teachers adequately, and they can easily master their children’s school performance, which is helpful with parents’ supervision and management. Teachers’ concern and care for their children will significantly enhance academic self-efficacy. According to the self-determination theory, enhancing self-efficacy can stimulate internal motivation and inhibit risky behaviors. Then it will be a disincentive effect on smartphone addiction.

To sum up, it can be concluded that family social capital is an important factor influencing students’ smartphone addiction. From the perspectives of both social capital within and both social outside family, parents with the former can have more communication with children, and positively familiarize with their children’s latest development of life and academic study, which is also a comprehensive supervision on children.

Parents with latter capital are able to communicate with teachers frequently and laterally grasp their children’s school performance. To a certain extent, they can effectively curb the smartphones addiction. Therefore, this study proposes the following hypotheses.

*H3a*: There is a negative correlation between social capital outside the family and smartphone addiction.

*H3b*: There is a negative correlation between social capital within family and smartphone addiction.

### 3.4 The effect of school climate on smartphone addiction

Person-environment Fit Theory points out that individuals are greatly affected by the environment, and a good environment atmosphere will generate positive results for individuals. If the theory of Person-environment Fit is used between school climate and college students it means that a positive school climate is crucial to the growth and development of students. To be specific, a positive school climate can enrich the inward spiritual and cultural world of college students, promote their integration into the college life, enhance their satisfaction and psychological health, promote the fundamental psychological needs, and avoid negative emotions such as loneliness and boredom. According to Self-determination Theory, if the fundamental psychological needs are satisfied, it can greatly stimulate students’ intrinsic motivation to actively seek self-growth and inhibit risky behaviors. On the contrary, if they are not met, students feel frustrated, anxious, and less motivated to strive, and they will resort to such risky behaviors as virtual worlds of online games and smartphones for the psychological satisfaction. It was found that positive school climate has a significantly negative predictor on students’ school risk behaviors (Zhu et al., 2015). Smartphone addiction has the characteristics of school risk behaviors, and thus positive school climate has a negative effect on their smartphone addiction. In particular, from the perspective of social psychological atmosphere, school climate is manifested in three aspects, namely teacher support, student support and autonomy opportunity. Teacher support and students support help to meet students’ relationship needs, while autonomy opportunity helps to meet students’ autonomy needs. When basic psychological needs are met, it can slow down the occurrence of risk behaviors (Sun et al., 2021). Some scholars have also declared that whether students’ behavior is normalized or random is influenced by the degree of teacher support and peer support (Bussemakers and Denessen, 2023; Zhao and Qin, 2021). Generally speaking, with the full support of teachers and peers, learning behavior should be in a normal state. At the same time, studies have supported that autonomous opportunities can reduce teenagers’ anxiety level (Way et al., 2007) and meet teenagers’ psychological needs (Way and Robinson, 2003). As a result, it can inhibit negative risk behaviors and avoid smartphone addiction to a certain extent.

Based on previous empirical studies, “Person-environment” Fit Theory, and Self-determination Theory, school climate is an important factor affecting students’ dependence on mobile phones. Meanwhile, from the perspective of social psychological atmosphere, this paper analyzes the impact of school climate on smartphone addiction from three aspects: teacher support, student support and autonomy opportunities, and proposes the following hypotheses:

*H4a*: There is negative correlation between teacher support and college students’ smartphone addiction.

*H4b*: There is negative correlation between peers support and college students’ smartphone addiction.

*H4c*: There is negative correlation between autonomy opportunities and college students’ smartphone addiction.

### 3.5 The interaction effect between school climate and family capital on smartphone addiction

Ecological Systems Theory states that schools and families, as micro-systems of individual development, independently influence individual development on the one hand, while at the same time there are interactions and connections (Bluteau et al., 2017). At the same time, Overlapping spheres of influence theory believes that the family and the school have the same goal and mission for the development of adolescents, and their “combined force” can play a multiplying effect on the development of adolescents (Galindo and Sheldon, 2012). The effect of their “synergy” on adolescent development can be twice as effective. From empirical studies, scholars have analyzed the interaction between family capital and the environment of the school in which the children are enrolled in order to influence the development of students (Yang, 2017).

From this, it can be seen that there is a positive interaction between school climate and family capital on undergraduate students’ behavior at school. Smartphone addiction, as a specific school behavior of undergraduate students, is inevitably subject to the combined effects of school climate and family capital. Therefore, this study formulates the hypotheses at the aggregate level:

*H5*: There is a positive interaction effect of school climate and family capital on undergraduate students’ smartphone addiction.

## 4 Date, variable, and model

### 4.1 Data source

This study adopted the principle of stratified sampling. In this study, the data of subjects were collected by using the electronic questionnaire on Wenjuanxing platform. Subjects are from 7 different types of universities in different cities of Shandong province, including normal, agriculture and forestry, medicine, science and technology and comprehensive universities. A total of 632 questionnaires were sent out, and 632 were collected. In this study, the questionnaires were screened and cleaned based on two criteria. First, reverse-coded items were examined; if the responses to reverse-coded items contradicted those to positively worded items, the questionnaire was considered invalid. Second, questionnaires with a repetition rate exceeding 80% (including 80%) were excluded. After back-to-back manual screening, 556 valid data were selected, and the effective recovery rate was 87.97%. The sample size of the data used in the research is comparatively large, the coverage is wide, and the reliability is convincing. The investigation contents cover smartphone addiction, family cultural capital, family social capital, family economic capital, school climate, gender, grade, household registration location, and the number of children in the family.

### 4.2 Variable selection and processing

In this study, the questionnaire was adapted from established and validated instruments. First, Cronbach’s α coefficient was calculated to assess the reliability of the questionnaire. Second, three experts in the field of education and thirteen university students were invited to evaluate the content validity. Items that were difficult to understand were revised accordingly.

#### 4.2.1 Dependent variable

The research takes the smartphone addiction of undergraduates as the dependent variable, and adopts smartphone addiction scale (SAS-SV) (Kwon et al., 2013), which is universally acknowledged in academia. The scale is composed with 10 items, using a five-point Likert scale (1: “strongly disagree” and 5: “strongly agree.”) The score of the total items was added up in the result analysis. The higher the score indicate the higher the level of the addiction. The Cronbach’s α coefficient of the scale used in the study is 0.83. According to the test basis of Hair et al on the reliability of the scale, if the Cronbach’s α coefficient is greater than 0.7, it can be accepted as a good consistency among items (Hair et al., 2009).

#### 4.2.2 Variable

The types of family cultural capital are embodied, objectified and institutionalized. Parents’ level of emphasis on education is used to measure family embodied cultural capital, the amount of book collection in the family is used to measure family objectified cultural capital, and the parents’ final official academic credentials is used to measure family institutionalized cultural capital (Yu et al., 2022). Family social capital can be classified into social capital within the family and outside the family. Family social capital is divided into social capital within the family and outside the family. On the one hand, Coleman’s family social capital theory in the field of pedagogy is referenced in this study (Coleman, 1988), and parent-child communication acts as a measure of social capital within the family. Then a five-point scale are created (1: “never” and 5: “very high”). On the other hand, parent-child teacher interaction acts as a measure of family social capital outside the family. Then a five-point scale is created (1: “never” and 5: “very high”). Family economic capital scale which is demonstrated by annual family income also draws from the questionnaire options compiled by Bahna (2018).

School climate, as a complex and multi-dimensional concept, is mostly measured from the perspectives of organizational atmosphere, social system environment, and social psychological atmosphere and so on. Among them, the scale was developed by Jia et al. (2009) and measured from a psychosocial climate perspective. It is subdivided by three dimensions of teacher support, peer support and opportunities for autonomy (Jia et al., 2009), which gains the popularity among Chinese youth groups with good reliability and validity. This research adopts the school climate questionnaire developed by Jia et al. (2009) for investigation. The questionnaire was divided into three dimensions, including faculty support, peer support and opportunities for autonomy. Totally there are 25 items, including 7 reverse scoring questions. This five-point Likert scale, ranging from “1” for “strongly disagree” to “5” for “strongly agree”. The high scale scores indicate the undergraduate’s university climate is superior. In this study, the Cronbach’s a coefficient of the total scale was 0.89, and the Cronbach’s a coefficients for the dimensions of faculty support, peer support, and opportunities for autonomy were 0.84, 0.80, and 0.87, respectively.

#### 4.2.3 Control variable

In order to avoid the interference of gender, grade, subject, only child and place of household registration on the study of undergraduates’ smartphone addiction, they are set as control variables. Gender is a dichotomous variable (0: “boy” and 1: “girl”). Grade is a quartile variable, which is treated by dummy variable, and freshman is the basic group; the place of household registration is divided into urban areas and townships (0: “urban areas” and 1: “townships”); the number of family children is divided into only child and non-only child (0: “only child” and 1: “non-only child”) (see Table 1).

**TABLE 1:** Explanation of the meaning of the variables.

Variables	Instructions
Implicit variable	
Smartphone addiction	Based on the short version of the Smartphone Addiction Scale (SAS-SV), scored on a five-point Likert scale, with 1 indicating “strongly disagree” and 5 indicating “strongly agree,” the item totals were used in the outcome analysis.
Independent variables	
Family embodied cultural capital	Using a five-point scale of parental importance of education, with 1 indicating no importance and 5 indicating high importance, in ascending order of importance
Families objectify cultural capital	Based on household book collections (excluding magazines and e-books)
Family institutionalized cultural capital	The highest education level of the parents is used, with 1 indicating elementary school and below, 2 indicating junior high school, 3 indicating senior high school (including secondary vocational specialties), 4 indicating bachelor’s degree (including higher vocational specialties), and 5 indicating postgraduate studies.
Intra-family social capital	Based on the level of interaction between parents and children, with 1 indicating never, 2 indicating less, 3 indicating average, 4 indicating more, and 5 indicating a lot.
Extra-family social capital	Based on the level of parent-counselor interaction, with 1 indicating never, 2 indicating less, 3 indicating average, 4 indicating more, and 5 indicating many
Household economic capital	Expressed in terms of annual household income
Teacher support	Based on the teacher dimension of the School Climate Scale developed by Jia et al., which consists of seven items scored on a five-point Likert scale, with 1 indicating “strongly disagree” and 5 indicating “strongly agree.”
Autonomy opportunity	Based on the autonomy opportunity dimension of the School Climate Scale developed by Jia et al., which consists of five items scored on a five-point Likert scale, with 1 indicating “strongly disagree” and 5 indicating “strongly agree.”
Student support	Based on the student-supported items of the School Climate Scale developed by Jia et al., there are 13 items with 7 reverse scored items on a 5-point Likert scale, with 1 indicating “Strongly Disagree” and 5 indicating “Strongly Agree”
Control variable	
Genders	0 for male, 1 for female
Number of children in the family	0 for only child, 1 for non-only child
Registered residence	0 for urban areas, 1 for townships
Grades	1 for freshman, 2 for sophomore, 3 for junior, and 4 for senior, operating with freshman set as the reference dummy variable

### 4.3 Descriptive statistics of variables

Prior to the descriptive statistical analysis of the variables, confirmatory factor analysis (CFA) was conducted to examine the degree of fit between the theoretical model and the collected data. The validity of the questionnaire was evaluated using confirmatory factor analysis (CFA). Convergent validity was assessed by calculating the average variance extracted (AVE) and composite reliability (CR). Structural validity was examined using the comparative fit index (CFI), Tucker–Lewis index (TLI), and root mean square error of approximation (RMSEA). The results showed that all AVE values were above 0.5, all CR values exceeded 0.7, the CFI and TLI were both greater than 0.9, and the RMSEA was below 0.08, indicating good validity.

Table 2 reports the descriptive statistics of relevant variable. It can be found that the average score of smartphone addiction is 30.42, which means that the smartphone addiction of undergraduates exceeds the medium level and deserves focus. Meanwhile, in terms of family economic capital, the survey data show that the family income gap is large and fluctuates significantly. it is necessary to focus on the material guarantee for students’ academic life from low-income families. As to family social capital, the social capital within the family is slightly higher than the medium level, while the social capital outside the family is lower than the medium level. The above results show that the communication between parents and tutors needs to be significantly enhanced, and the communication between parents and children also needs to be further improved. In terms of family cultural capital, the final official academic credentials is between junior high school and high school (secondary vocational education), which is related to the fact that the parents of the subjects live in an era that belongs to the elite stage of higher education. Generally speaking, the college enrollment rate is still low. At the same time, the parents’ focus on education is close to “relatively important.” The average level of family book collection reaches 86.31 books, but the book collection gap is more significant. In terms of school climate, teacher support, opportunities for autonomy and student support all reach a slightly above-medium level.

**TABLE 2:** Descriptive statistics of kernel variable.

Variable	Observation	Mean ± SD	SD	Min.	Max.
Smartphone addiction	556	30.42 ± 0.29	6.85	10	50
Family economic capital	556	13.88 ± 1.88	44.43	0	1,000
Social capital within the family	556	3.35 ± 0.04	0.96	1	5
Social capital outside the family	556	2.63 ± 0.05	1.08	1	5
Family institutionalized cultural capital	556	2.74 ± 0.04	0.92	1	5
Family embodied cultural capital	556	3.67 ± 0.04	1.04	1	5
Family objectified cultural capital	556	86.31 ± 7.12	167.82	3	3000
Teacher support	556	25.05 ± 0.17	4.09	7	35
Autonomy opportunity	556	18.07 ± 0.13	3.01	5	25
Student support	556	53.06 ± 0.27	6.27	35	65

## 5 Empirical analysis

The following two research are worthy of further systematic and in-depth study. Firstly, whether there are differences in the degree of smartphone addiction due to demographic variables. Secondly, whether the influence of internal factors of family capital and school climate on undergraduates’ smartphone addiction are consistent with the hypotheses Therefore, the difference analysis and influencing factor analysis of undergraduates’ smartphone addiction from the aspects of family capital and school climate are the research emphasis.

### 5.1 The difference analysis of smartphone addiction

The difference analysis of smartphone addiction is mainly examined from the aspects of gender, number of children in the family, place of household registration and grade through Independent Sample *t*-test and One-way Analysis of Variance. The results shown in Table 3 indicate that the degree of smartphone addiction of female students is significantly higher than that of male students, which confirms the conclusions of previous studies (Yang et al., 2018). Further analysis shows that female students are more sensible to pressure than male students and are more likely to have addicted behaviors (Rospenda et al., 2008). Therefore, under stressful conditions, female college students may be more likely to become addicted on mobile phones. If taking factor of the household registration location into consideration, it can be found that the degree of smartphone addiction of urban students is lower than that of rural students, but it is not statistically significant. From the perspective of the number of children in a family, the degree of addiction on mobile phones of only children is significantly lower than that of non-only children, which is consistent with the Resource dilution model. The model means the development of each child depends on family resources and their distribution. As the number of children in a family increases, the degree of addiction on mobile phones of non-only children is significantly lower than that of non-only children (Downey, 2001). The resources that parents provide to each individual are also “diluted.” In other words, parents’ energy and time on caring children will be dispersed. As a result, the degree of psychological needs of children will be affected, and may turn to smartphones addiction. As for the grade, there are significant differences in the degree of mobile phone addiction among different grades, which is consistent with the conclusions of relevant studies (Tian et al., 2021). For instance, freshmen have the task of adapting to college life, and seniors have to think about the task of employment. In addition, the aim of talent development is also different among different grades, so the possibility of smartphone addiction is different to a certain extent.

**TABLE 3:** Mobile phone dependence difference analysis.

Variable		Mean ± SE	SD	*P*
Gender	Male (*n* = 146)	28.71 ± 0.631	7.629	0.001
	Female (*n* = 410)	31.03 ± 0.319	6.457	0.001
Household registration location	Urban (*n* = 218)	29.97 ± 0.466	6.876	0.21
	Rural (*n* = 338)	30.72 ± 0.372	6.834	0.21
Num. of children in the family	Only-child (*n* = 218)	30.72 ± 0.372	7.13	0.022
	Non-only-child (*n* = 338)	29.95 ± 0.466	6.68	0.022
Variable		Mean ± SD	SD	*P*
Grade	Freshman (*n* = 321)	30.36 ± 0.388	6.953	0.025
	Sophomore (*n* = 135)	31.95 ± 0.563	6.538	0.025
	Junior (*n* = 31)	27.03 ± 1.413	7.868	0.025
	Senior (*n* = 69)	31.20 ± 0.742	6.161	0.025

### 5.2 Analysis of influencing factors of smartphone addiction from the perspective of family and school field

The choice of the empirical regression research method is related to the type structure of the explanatory variables and the purpose of the study. The smartphone addiction variable is a continuous variable. Meanwhile, this research aims to explore the influence of family capital and school climate on undergraduate students’ smartphone addiction, so the stepwise regression method was employed to examine the factors influencing undergraduate students’ smartphone addiction status from both family and school perspectives. Finally, curbing smartphone addiction can be achieved due to the cooperation between home and school.

In order to explore the influencing factors of undergraduates’ smartphone addiction from the dual perspectives of home and school, we adopt the form of stepwise regression, introduce four groups of variables on the basis of control variables, and establish four regression models. In Model 1, three kinds of family cultural capital variables are added on the basis of control variables. It is found that family embodied cultural capital variables and family objective cultural capital variables have significant negative effects on undergraduates’ smartphone addiction. On the contrary, family institutionalized cultural capital variables have no significant effects on undergraduates’ smartphone addiction, so H1a and H1b are verified, while H1c is rejected. On the basis of Model 1, the variables of social capital within the family and social capital outside of the family are added in Model 2. It is found that both variables have a significant negative effect on undergraduates’ smartphone addiction, so H2a and H2b are verified. Based on Model 2, family economic capital variables are added in Model 3. It is found that family economic capital impact on undergraduates’ smartphone addiction is not significant, so H3 is rejected. Three dimensions of school climate are added in Model 4 on the basis of Model 3. It is found that teacher support variables and autonomy opportunity variables have no significant effect on undergraduates’ smartphone addiction, while student support has a significant negative impact on undergraduates’ smartphone addiction. Consequently, H4a and H4b are rejected, and H4c is verified. Finally, on this basis, drawing on the econometric model of the joint role of Julia Ogg and Christopher J. Anthony, the effect of home-school association on undergraduate students’ smartphone addiction was analyzed by adding the interaction terms of family capital and school climate indicators to the stepwise regression model (Ogg and Anthony, 2020) and it was found that the coefficients of the interaction terms of family-internal social capital, family-extra-familial social capital, and student support were −0.132 (*p* < 0.001), −0.108 (*p* < 0.001), that is, there is a mutual substitution effect, and the coefficients of the interaction terms of family embodied cultural capital, family objectivized cultural capital and student support are 0.104, 0.002 (*p* < 0.01), which show a mutually reinforcing effect, and H5 is partially verified (see Table 4).

**TABLE 4:** Regression results of family capital and school climate on undergraduates’ smartphone addiction.

	(1)	(2)	(3)	(4)
Variable	Model 1	Model 2	Model 3	Model 4
	Smartphone addiction	Smartphone addiction	Smartphone addiction	Smartphone addiction
Family embodied cultural capital	−0.675^***^	Regression results of family 0.398^***^	Regression results of family 0.399^***^	Regression results of family 0.379^***^
Standard error	(0.198)	(0.19)	(0.19)	(0.192)
Families objectify cultural capital	Regression results of family 0.191^***^	Regression results of family 0.119^***^	Regression results of family 0.12^***^	Regression results of family 0.109^***^
Standard error	(0.001)	(0.001)	(0.001)	(0.001)
Family institutionalized cultural capital	0.044	0.023	0.021	0.025
Standard error	(0.252)	(0.202)	(0.202)	(0.20)
Intra-family social capital		Regression results of family 0.177^***^	Regression results of family 0.176^***^	Regression results of family 0.172^***^
Standard error		(0.19)	(0.19)	(0.195)
Extra-family social capital		Regression results of family 0.406^***^	Regression results of family 0.406^***^	Regression results of family 0.37^***^
Standard error		(0.179)	(0.179)	(0.187)
Household economic capital			0.025	0.02
Standard error			(0.004)	(0.004)
Teacher support				Regression results of family 0.40
Standard error				(0.047)
Autonomy opportunities				0.019
Standard error				(0.057)
Student support				Regression results of family 0.088^***^
Standard error				(0.029)
Constant	46.502^***^	50.934^***^	50.828^***^	55.544^***^
Standard error	(1.221)	(1.044)	(1.049)	(1.794)
Sample size	556	556	556	556
R2	0.548	0.712	0.712	0.720

***p < 0.001, and standard errors are in parentheses.

## 6 Conclusion and suggestion

### 6.1 Proposals

First, focus on categorizing responses to smartphone addiction. Gender, grade, number of children in the family have significant difference on smartphone addiction, which requires us to appropriately address undergraduates’ mobile phone addiction and take countermeasures based on different influencing factors. From the perspective of gender, female students are significantly addicted on smartphone than male students. Schools should pay more attention to female students, and the rational use of mobile phones for girls should be promoted by persuasion. More activities should be organized for female students to attract them out of smartphone addiction. From the perspective of grade, the one-way variance shows that the degree of smartphone addiction among undergraduates in different grades is significantly different, so it is necessary to focus on different grades according to their situations. To be specific, the freshman year is the adjustment period for new students, and university should hold different activities to advocate the rational smartphone use according to their physical and mental conditions and to enrich their spare time. Sophomore year is the time when the excitement wears off and tasks are limited, so students should be encouraged to make career planning and avoid smartphone addiction. The junior year is a school year for many colleges and universities to arrange more courses, which reduces the dependence on smart phones to a certain extent. During the senior year, students are confronted with the pressure of further education and employment. As the stress-coping model depicts, the heavy pressure will greatly increase the individual’s sense of material craving, and even cause material dependence and addiction, such as smartphone addiction (Jeong et al., 2016). Schools should adopt various ways to relieve students’ pressure. In terms of the number of children in a family, the degree of mobile phone addiction of only children is significantly lower than that of non-only children, which is different from the social cognition about only children. However, the theory of resource dilution confirms that the more children in a family, the more time and frequency of communication between parents and children will be dispersed, which may be the reason why non-only children cannot receive adequate communication and supervision. Because their psychological needs cannot be met, they then turn to smartphones. In other words, we should understand the “dilution of love” of non-only children, get rid of the stereotype, and give non-only children enough care.

Second, making good use of family capital statutes smartphone addiction. Through the regression test of specific family capital and college students’ smartphone addiction, it can be found that family embodied capital, family objectified cultural capital, social capital within the outside the family are negatively correlated with smartphone addiction. However, family economic capital and family institutionalized capital have no significant effect on smartphone addiction. Therefore, the following suggestions are put forward:

Correctly understand and rationally use family economic capital. Family economic capital is a double-edged sword. High family economic capital can provide sufficient material support for students, but it also means that parents spend more time on work and less time on children’s communication. To avoid the smartphones addiction, we should be aware that family economic capital is not omnipotent, and rational use of family economic capital can provide necessary material support for school children, but we should not also neglect communication with children due to limited time. Therefore, parents should keep the balance between working hours and kids companionship.

Benign habitus should be transmitted through family embodied and objectified cultural capital. Social reproduction theory suggests that children’s school behaviors are influenced to some extent by family heritage factors, such as family embodied cultural capital, family objectified cultural capital, and family institutionalized cultural capital. Family institutionalized cultural capital is static in nature. If they are not involved in the avoidance of students’ smartphone addiction, and kept at home as a paper diploma, it is difficult to have the ideal effect. This is also the reason why the family cultural capital cannot significantly affect smartphone addiction. Thus, it is essential to develop the concept of attaching great importance to education. The concept of attaching importance to education in family culture should be fully constructed to achieve the effect of educational consciousness guiding educational actions, and further avoid college students’ addiction on smartphones. At the same time, the family cultural atmosphere should be created through the family book collection, and the family cultural habits at the material level should be implicitly transferred to the core, and the idea of valuing education should be implemented through various opportunities in daily life, so that college students can “inherit” the habitus and disposition to avoid the smartphone addiction.

Make good use of family social capital to enhance parental participation in education. The degree of communication between parents and children is a form of social capital within the family, and the degree of communication between parents and teachers is a form of social capital outside the family. The research confirms that social capital outside the family and social capital inside the family are significantly negatively related to the undergraduates’ smartphone addiction, which once again confirms that the more abundant social capital outside the family, it is easier to form a supportive community. Consequently, it is convenient for parents and teachers to exchange information about children’s learning and life and to supervise students’ behavior at school effectively. To this end, parents should strengthen the communication with tutors in time about the students’ latest development in school from the perspective of social capital outside the family, communication. From the perspective of social capital within the family, parents should take the initiative to communicate and interact with children, show their care, and be alert to the negative impact of bad information and electronic games on their children’s academic activities. At the same time, parents should spend more time with children and improve the quality of companionship, and generously support their children’s studies in material and emotional aspects.

Third, emphasis is placed on creating a supportive climate environment for students. Similar studies have shown that school climate plays a role in the process of college students’ mobile phone dependence (Tang et al., 2024). Education is the process of systematic socialization of the younger generation. University should provide a field space for students’ socialization. In this field, whether their campus behavior, as an external manifestation in the process of students’ socialization, are regulated or dissociated will be influenced by university atmosphere to a certain extent, especially by peers influence. At the same time, through empirical test, student support has a significant negative impact on undergraduates’ smartphone addiction, while teacher support and autonomy opportunities have no significant effect. Therefore, it is found that the influence of student support is indeed “high reference” and “high affinity,” while according to the survey data, the degree of teacher support and autonomy opportunities is only slightly higher than the medium degree. This is why it is insignificant. Therefore, it is necessary to create an atmosphere of mutual care among students to meet the basic psychological needs of undergraduates and avoid risky behaviors caused by negative emotions such as loneliness and boredom, or they may turn to other environments (such as smartphone addiction, etc.) to seek the satisfaction of basic psychological needs.

Fourthly, we should emphasize cooperation between families and schools and control the degree of family participation. Research reached similar conclusions that both family factors and school factors had an impact on smartphone addiction(Roser et al., 2016).For example, the Opinions on Family-School-Society Collaborative Parenting Mechanisms issued by the Ministry of Education and other thirteen departments emphasize that families and schools are important places for parenting, and that college students’ smartphone addiction is an important part of parenting that deserves the joint attention of families and schools. The study found that family embodied cultural capital, family objective cultural capital and student support show a mutually reinforcing relationship, which means that we should deepen the construction of students’ mutual help atmosphere and the interactive integration of family embodied cultural capital and family objective cultural capital, incorporate students’ mutual help habitus into the construction of family culture, and respect each other’s family cultural habitus in the mutual help of students; intra-family social capital, extra-family social capital and student support show a mutual substitution role; intra-family social capital, extra-family social capital and student support show a mutual substitution role. Social capital within the family, social capital outside the family and student support present mutual substitution, meaning that the moderate use of family social relations and student pathways to supervise and manage their children, avoiding excessive care and attention, or else it will produce the opposite effect. At the same time, optimize the way of home-school cooperation, clarify the role of parents in increasing the management of school education, do not lack of position, do not overstep the position, and improve the level of home-school cooperation.

### 6.2 Limitations and future prospects

The article explored the influencing factors of undergraduate students’ smartphones from the dual perspective of home and school, and made some valuable conclusions, but there are still some limitations. First, the study obtained cross-sectional data through questionnaires, which is difficult to dynamically reflect the dynamic changes of undergraduates’ smartphone addiction. Future research can be based on a combination of cross-sectional and longitudinal studies to elucidate the dynamic changes of undergraduate smartphone addiction. Second, the factors affecting undergraduates’ smartphone addiction are diversified, and the article focuses on factors such as family capital and school climate, which to a certain extent misses some factors, and does not consider the mediating mechanism in exploring the influencing factors of undergraduates’ smartphone addiction. Finally, future research can further explore the mechanism of home-school synergy from multiple angles under the mechanism of home-school independence and collaboration, in order to clarify the specific aspects of home-school synergy.

## Data Availability

The original contributions presented in the study are included in the article/supplementary material, further inquiries can be directed to the corresponding author.
